# Supplementary material for: SNHG15-Mediated Localization of Nucleolin at the Cell Protrusions Regulates CDH2 mRNA Expression and Cell Invasion
Source: Int J Mol Sci. 2023 Oct 26;24(21):15600. doi: 10.3390/ijms242115600 (PMC10650932; doi:10.3390/ijms242115600)
Supplement: Supplementary file 1 [file ijms-24-15600-s001.zip › ijms-2600889-supplementary.pdf]

Article

SNHG15 mediated localization of nucleolin at the cell protrusions regulates CDH2 mRNA expression and cell invasion

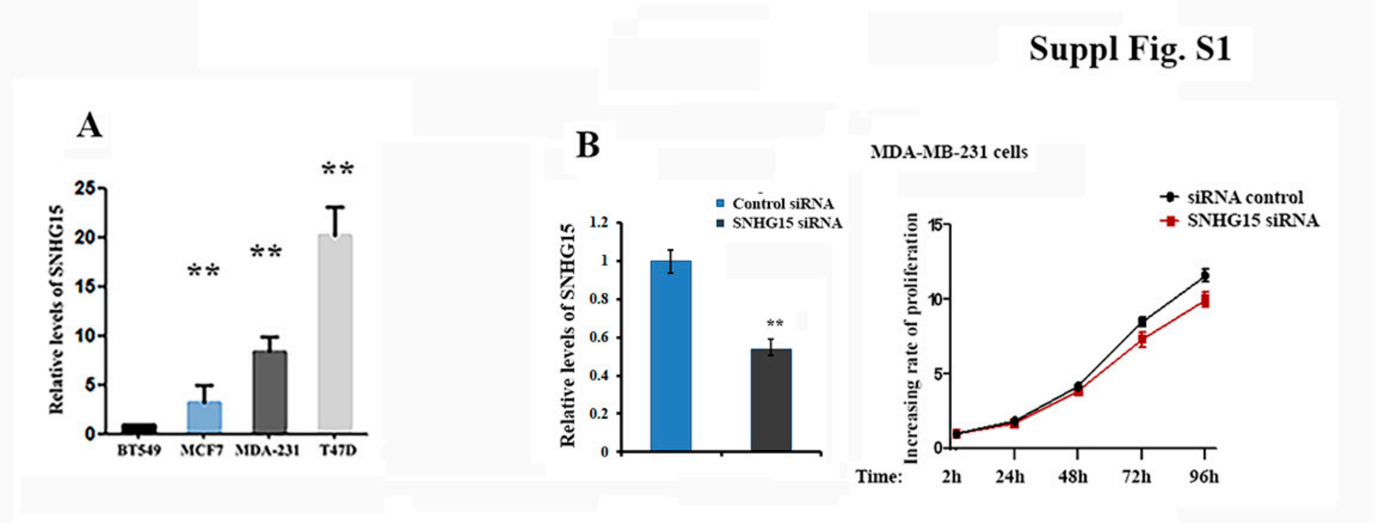

Figure S1. SNHG15 is one of the lncRNAs that associates with IMP1 in breast cancer cells. (A) RT-qPCR indicates the relative levels of endogenous SNHG15 in four breast cancer cell lines. (B) Cell proliferation assays were performed in MDA-MB-231 cells, which showed that knocking down SNHG15 expression by siRNA (left panel) decreased cell growth potential (right panel).

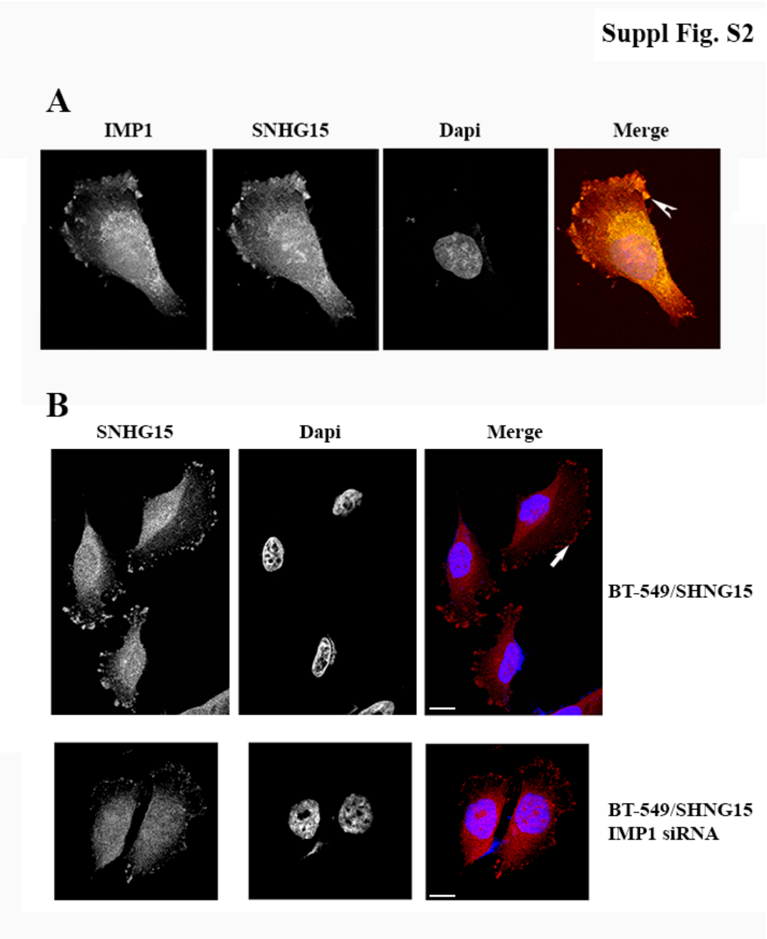

Figure S2. IMP1 directs the localization of SNHG15. (A) FISH and IF staining showed that SNHG15 and IMP1 were colocalized at the cell protrusions of BT-549 cells (pointed by the arrowhead). (B) FISH was performed in BT-549 cells expressing ectopic SNHG15. Results indicated that knocking down IMP1 expression by siRNA decreased protrusion localization of SNHG15. .

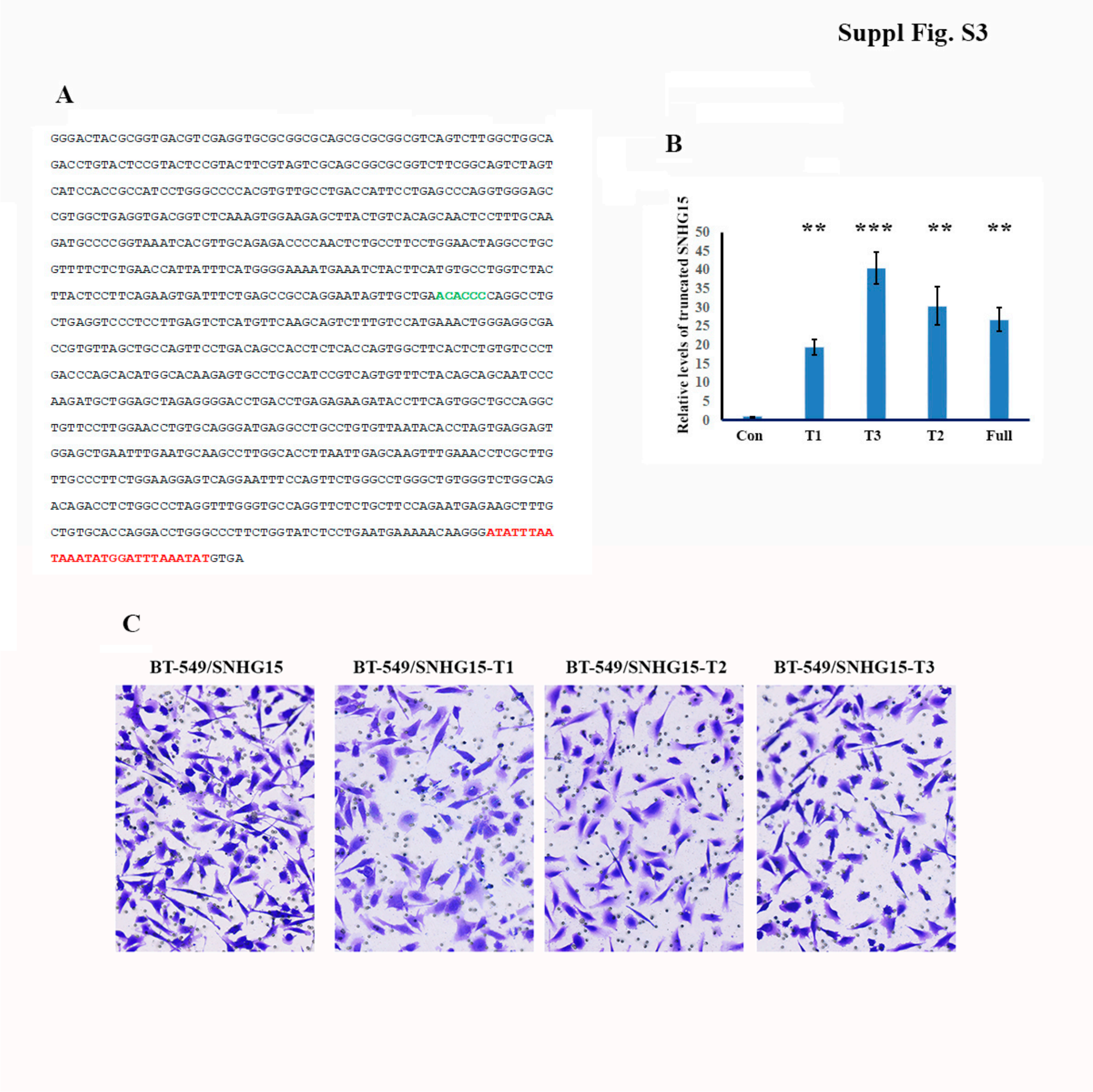

Figure S3. Nucleotide sequence of SNHG15 and the effects of its ectopic expression on cell invasion. (A) Nucleotide sequences of SNHG15 are shown. The green-colored 'ACACCC' is the motif for IMP1 binding and the red-colored sequence is a predicted region potentially for nucleolin binding. (B) Stable BT-549 cell lines expressing truncated SNHG15 were established. Relative levels of truncated SNHG15 in stable cell lines were measured by RT-qPCR. (C) Representative images of the transwell assays in cells expressing SNHG15 full-length or truncated SNHG15 variants. .

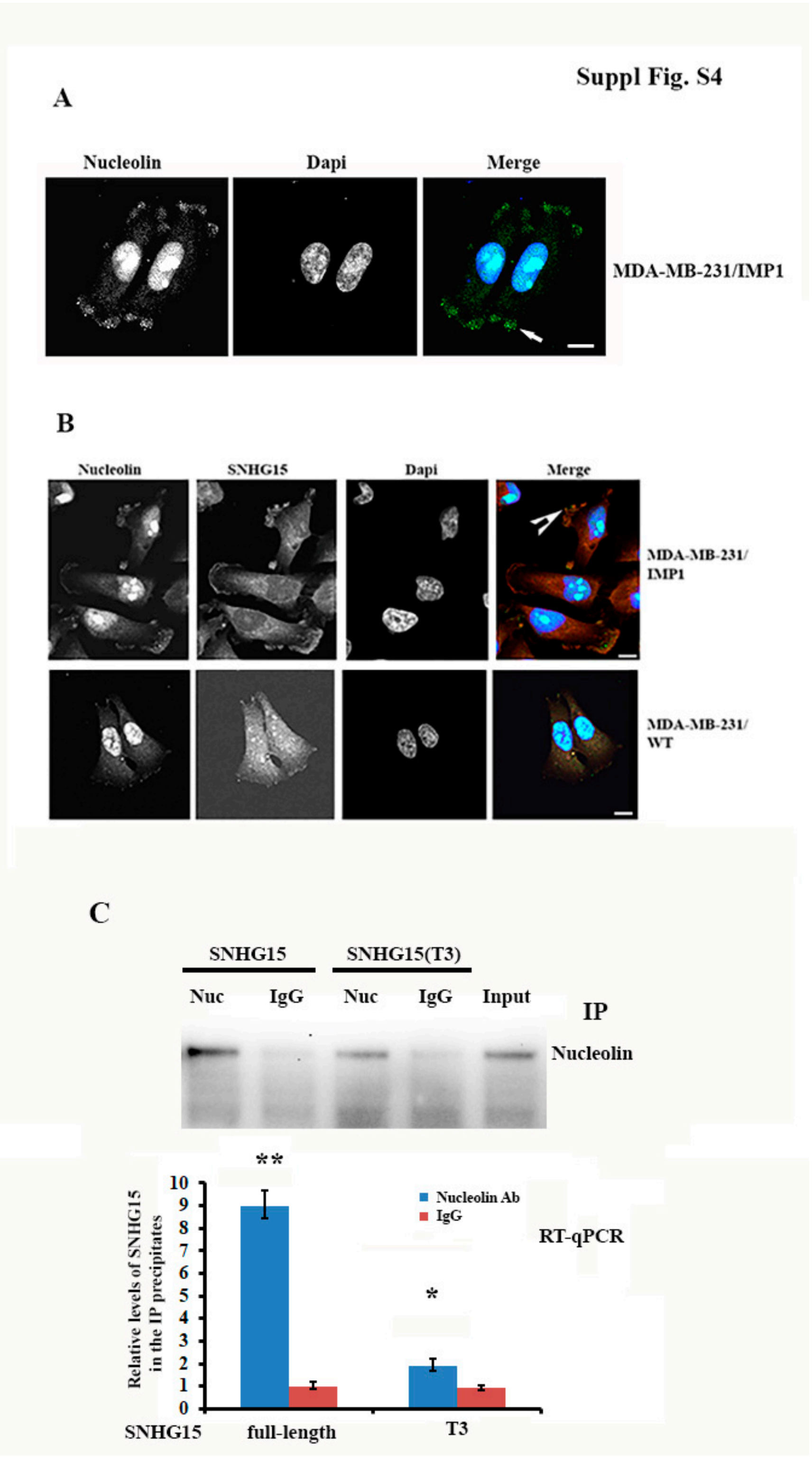

Figure S4. IF and FISH show the localization of nucleolin and SNHG15. (A) IF indicates that nucleolin was accumulated in MDA-MB-231 cells expressing ectopic IMP1. The arrowhead indicates detected nucleolin. Scale bar: 10 μm. (B) FISH and IF double staining assays showed that colocalization of nucleolin and SNHG15 at the cell protrusions was also relied on IMP1. Scale bar: 10 μm. (C) RIP experiments using nucleolin antibodies were performed to detect the binding of nucleolin to full-length and truncated T3 SNHG15 (refer to the Fig. 3A). Western blots (upper panel) and RT-qPCR (lower panel) indicated that truncated T3 SNHG15 does not bind to nucleolin.

Suppl Fig. S5

A

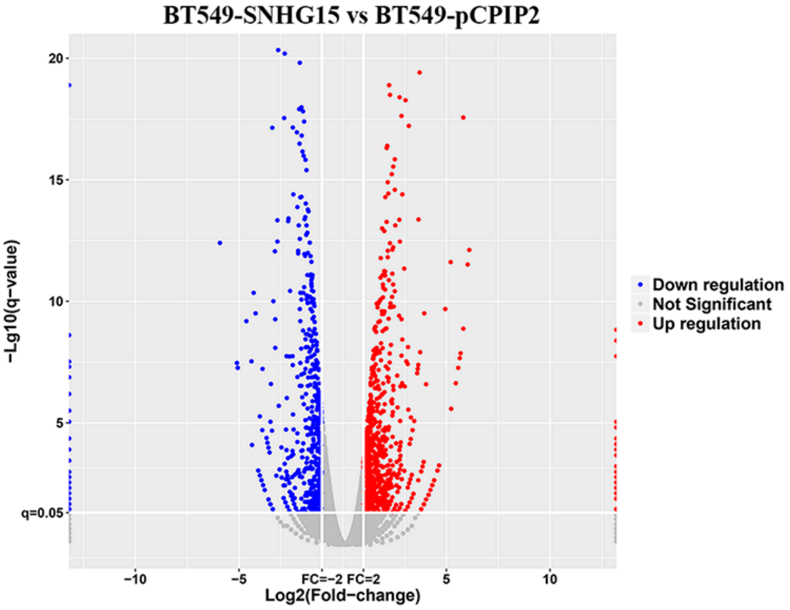

B

gene ID	gene name	description	log2FC
ENSG00000170558	CDH2	cadherin 2	2.51596
ENSG00000076554	TPD52	tumor protein D52	2.437499
ENSG00000105825	TFPI2	tissue factor pathway inhibitor 2	-1.21677
ENSG00000100234	TIMP3	TIMP metallopeptidase inhibitor 3	-2.94468
ENSG00000170873	MTSS1	MTSS I-BAR domain containing 1	-2.82187
ENSG00000120279	MYCT1	MYC target 1	-1.7165

C

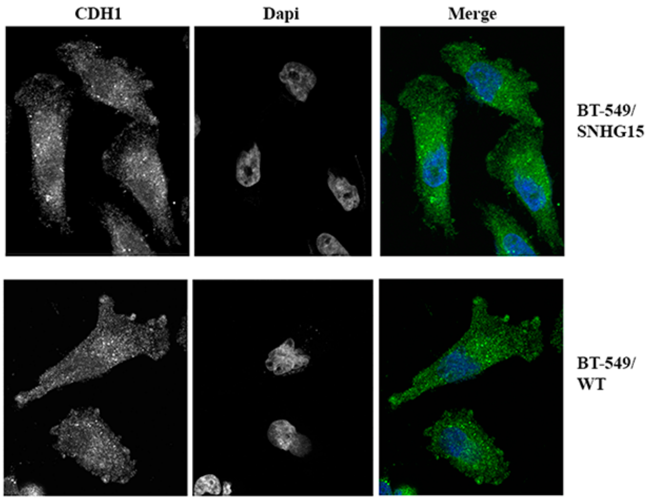

Figure S5. IF shows the cellular distribution of CDH1 protein. (A)A volcano plot with differentially expressed genes was shown in BT549-SHNG15 cells compared with BT549-PCPI2 cells. (B) A table listed six significant individual transcripts that were up-regulated or down-regulated in BT-549 cells when SNHG15 was expressed. (C) IF assays were performed using antibodies against human CDH1 (E-cad) proteins. Results show that CDH1 protein was widely expressed in both BT-549 WT and SNHG15-expressing cells.

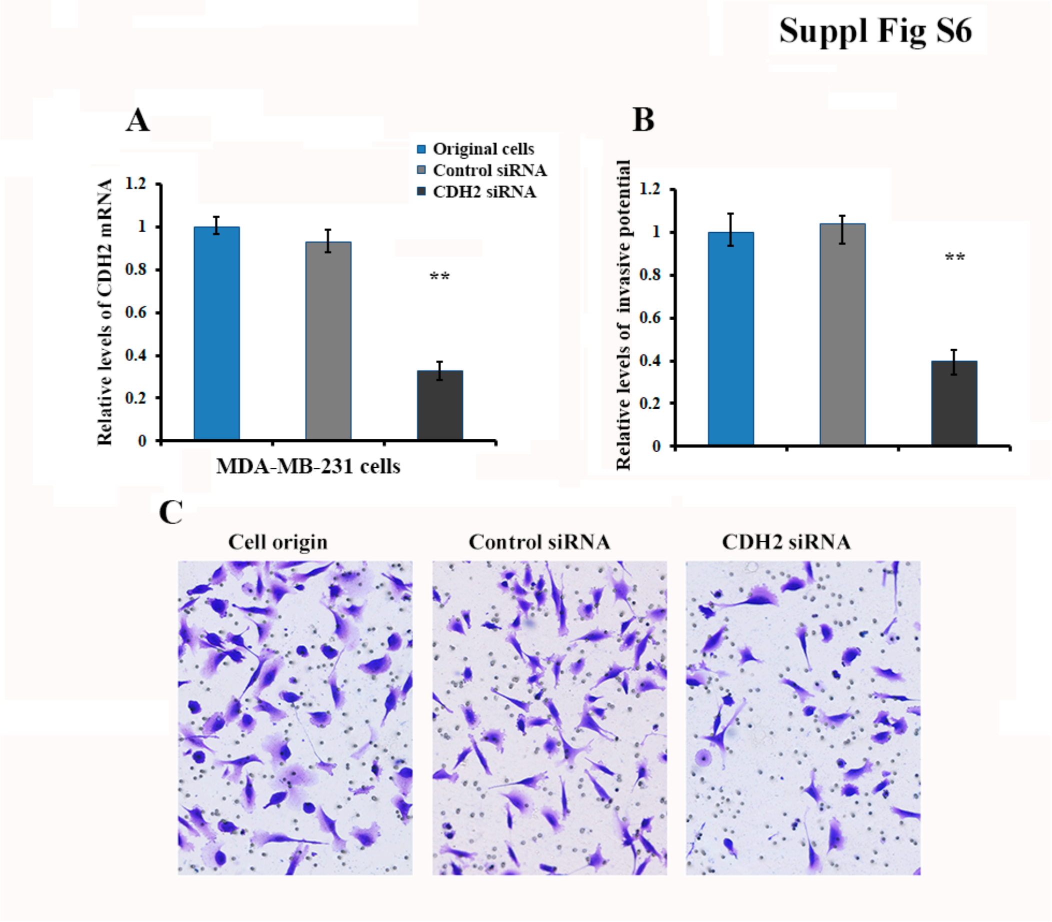

Figure S6. Knocking down CDH2 reduced invasion of MDA-MB-231 cells. (A) Relative levels of CDH2 mRNA were measured by RT-qPCR after cells were treated with siRNA for 48 hrs. (B) Transwell assays indicated that knocking down CDH2 significantly reduced cell invasive potential. (C) Representative images indicate the transwell assays in CDH2 knocking down and control cells.

Table S1. Identification of IMP1 associated lncRNAs by RNA-seq. IMP1 was precipitated and its associated lncRNAs were identified by RNA-seq. Part of lncRNAs with higher affinity to IMP1 were listed in the table.

Gene ID	Gene name	Gene description	Fold changes: IMP1 Ab/IgG
ENSG00000232956	SNHG15	small nucleolar RNA host gene 15	721370
ENSG00000228288	PCAT6	prostate cancer associated transcript 6	76176.2
ENSG00000130600	H19	H19, imprinted maternally expressed transcript	418503
ENSG00000256040	PAPPA-AS1	PAPPA antisense RNA	19730
ENSG00000182165	TP53TG1	TP53 target 1	756405
ENSG00000197308	GATA3-AS1	GATA3 antisense RNA 1	626405
ENSG00000254389	RHPN1-AS1	RHPN1 antisense RNA 1	1526700
ENSG00000227617	CERS6-AS1	CERS6 antisense RNA 1	2.544540491
ENSG00000272695	GAS6-AS2	GAS6 antisense RNA 2	164341
ENSG00000176124	DLEU1	Deleted in lymphocytic leukemia 1	331798
ENSG00000245694	CRNDE	Colorectal neoplasia differentially expressed	8.0847
ENSG00000226950	DANCR	Differentiation antagonizing non-protein coding RNA	2.87622373

Table S2. Potential proteins associated with SNHG15. SNHG15 was pulled down. Proteins associated with SNHG15 were isolated and analyzed by spectrometry assays. Part of the proteins potentially associated with SNHG15 were listed.

Protein name	Accession	Avg. Mass	Description
PAIRB	Q8NC51	44965	Plasminogen activator inhibitor 1 RNA-binding protein 1
NUCL	P19338	76615	Nucleolin
PABP1	P11940	70671	Polyadenylate-binding protein 1
DDX5	P17844	69148	Probable ATP-dependent RNA helicase DDX5
DDX1	Q92499	82432	ATP-dependent RNA helicase DDX1
FUS	P35637	53426	RNA-binding protein FUS
SF3B2	Q13435	100228	Splicing factor 3B subunit 2
ROA1	P09651	38747	Heterogeneous nuclear ribonucleoprotein A1
PRP8	Q6P2Q9	273599	Pre-mRNA-processing-splicing factor 8
VIGLN	Q00341	141455	Vigilin
EHD2	Q9NZN4	61162	EH domain-containing protein 2
MYH9	P35579	226530	Myosin-9
DDX41	Q9UJV9	69838	Probable ATP-dependent RNA helicase DDX41
VDAC3	Q9Y277	30659	Voltage-dependent anion-selective channel protein 3
GTF2I	P78347	112416	General transcription factor II-I
HNRPF	P52597	45672	Heterogeneous nuclear ribonucleoprotein F
ILF2	Q12905	43062	Interleukin enhancer-binding factor 2
CCAR2	Q8N163	102902	Cell cycle and apoptosis regulator protein 2
IQGA1	P46940	189251	Ras GTPase-activating-like protein IQGAP1
HNRPM	P52272	77516	Heterogeneous nuclear ribonucleoprotein M
AP2A1	O95782	107546	AP-2 complex subunit alpha-1
TFR1	P02786	84871	Transferrin receptor protein 1

Table S3. Primers, siRNAs and FISH probes used in the study.

Primers	Sequences
GAPDH Forward	GAGTCAACGGATTTGGTCGT
GAPDH Reverse	TGGGATTTCCATTGATGACA
SNHG15 Forward	TCTAGTCATCCACCGCCATC
SNHG15 Reverse	AGAGAAAACGCAGGCCTAGT
SNHG15 truncate F	TGACAGCCACCTCTCACCAGTG
SNHG15 truncate R	CAGCCACTGAAGGTATCTTCTC
CDH2 Forward	TGCGGTACAGTGTAACTGGG
CDH2 Reverse	GAAACCGGGCTATCTGCTCG
TPD52 Forward	AACAGAACATTGCCAAAGGGTG
TPD52 Reverse	TGACTGAGCCAACAGACGAAA
TFPI-2 Forward	GTCCCAAGAAGACAAAGTCGCA
TFPI-2 Reverse	GTGGTCTCCAACCCACAATGTC
TIMP3 Forward	TTCCTCCTTTGGGCATCT
TIMP3 Reverse	ACACTTGGGTGCCATCTT
MYCT1 Forward	GCCAGAAAACTTTTGGGAGGA
MYCT1 Reverse	ATCCAGTTCTGTTGAGGCCG
MTSS1 Forward	ACATGGCCACCAACACACGTG
MTSS1 Reverse	CGAAAACTGCCTCAGCTTGGCT
**siRNAs**	**Sequences**
For CDH2 mRNA-1	UUUAUCUCUAUCAGACCUGTT
For CDH2 mRNA-2	UUGUCAACAUGGUACCGGCTT
For SNHG15-1	UUUCAUGGACAAAGACUGCTT
For SNHG15-2	UAGGUGUAUUAACACAGGCTT
**FISH probes**	**Sequences**
For SNHG15 RNA-1	gtctctgcaacgtgatttac
For SNHG15 RNA-2	ggttcagagaaaacgcaggc
For CDH2 mRNA-1	tggagttttctggcaagttg
For CDH2 mRNA-2	acactgtaccgcagtgaaag
For CDH2 mRNA-3	acctggtgtaagaactcagg
